# Supplementary material for: Severe pulmonary complications after cytoreductive surgery and hyperthermic intraperitoneal chemotherapy are common and contribute to decreased overall survival
Source: PLoS One. 2021 Dec 28;16(12):e0261852. doi: 10.1371/journal.pone.0261852 (PMC8714091; doi:10.1371/journal.pone.0261852)

| **SUPPLEMENTARY FILE - REGRESSION MODELS IN MANUSCRIPT**  **Final model on risk factors for severe pulmonary complications after CRS and HIPEC. Includes regression coefficients, their standard errors, p-values, odds ratios and confidence intervals.**   \|  \| \| \| \| \| \| \| \| \| \| \| --- \| --- \| --- \| --- \| --- \| --- \| --- \| --- \| --- \| --- \| \|  \| \| B \| S.E. \| Wald \| df \| Sig. \| Exp(B) \| 95% C.I.for EXP(B) \| \| \| Lower \| Upper \| \| Step 1^a^ \| Diaphragmatic peritonectomy \| ,603 \| ,378 \| 2,551 \| 1 \| ,110 \| 1,828 \| ,872 \| 3,832 \| \| Full thickness diaphragmatic injury and/or diaphragmatic resection \| 1,685 \| ,312 \| 29,104 \| 1 \| ,000 \| 5,393 \| 2,924 \| 9,948 \| \| PCI \| ,019 \| ,017 \| 1,294 \| 1 \| ,255 \| 1,020 \| ,986 \| 1,054 \| \| Liver resection \| ,455 \| ,327 \| 1,941 \| 1 \| ,164 \| 1,576 \| ,831 \| 2,991 \| \| Age \| ,000 \| ,000 \| 2,793 \| 1 \| ,095 \| 1,000 \| 1,000 \| 1,000 \| \| Smoking \| ,473 \| ,315 \| 2,256 \| 1 \| ,133 \| 1,605 \| ,866 \| 2,975 \| \| Constant \| -3,599 \| ,544 \| 43,700 \| 1 \| ,000 \| ,027 \|  \|  \| \|  \| \| \| \| \| \| \| \| \| \| |
| --- | --- | --- | --- | --- | --- | --- | --- | --- | --- | --- | --- | --- | --- | --- | --- | --- | --- | --- | --- | --- | --- | --- | --- | --- | --- | --- | --- | --- | --- | --- | --- | --- | --- | --- | --- | --- | --- | --- | --- | --- | --- | --- | --- | --- | --- | --- | --- | --- | --- | --- | --- | --- | --- | --- | --- | --- | --- | --- | --- | --- | --- | --- | --- | --- | --- | --- | --- | --- | --- | --- | --- | --- | --- | --- | --- | --- | --- | --- | --- | --- | --- | --- | --- | --- | --- | --- | --- | --- | --- | --- | --- | --- | --- | --- | --- | --- |
|  |
| **Overall model goodness**-**of-fit:**   \| **Hosmer and Lemeshow Test** \| \| \| \| \| --- \| --- \| --- \| --- \| \| Step \| Chi-square \| df \| Sig. \| \| 1 \| 1,902 \| 8 \| ,984 \| |

| **Final model on overall survival after CRS and HIPEC. Includes regression coefficients, their standard error, p-values, hazard ratios and confidence intervals** | | | | | | | | |
| --- | --- | --- | --- | --- | --- | --- | --- | --- |
|  | B | SE | Wald | df | Sig. | Exp(B) | 95,0% CI for Exp(B) | |
|  |  |  |  |  |  |  | Lower | Upper |
| No complication |  |  | 7,551 | 3 | ,056 |  |  |  |
| PPCs ≥3 | ,271 | ,256 | 1,113 | 1 | ,291 | 1,311 | ,793 | 2,167 |
| Non-pulmonary complications ≥3 | ,230 | ,367 | ,392 | 1 | ,531 | 1,259 | ,613 | 2,586 |
| PPCs and non-pulmonary complications ≥3 | ,827 | ,315 | 6,867 | 1 | ,009 | 2,285 | 1,232 | 4,241 |
| BMI 18.5-25 |  |  | 12,516 | 3 | ,006 |  |  |  |
| BMI > 18.5 | 1,544 | ,535 | 8,320 | 1 | ,004 | 4,683 | 1,640 | 13,369 |
| BMI >25 – 30 | -,320 | ,187 | 2,918 | 1 | ,088 | ,726 | ,503 | 1,048 |
| BMI > 30 | -,093 | ,234 | ,158 | 1 | ,691 | ,911 | ,576 | 1,442 |
| CC score | -,542 | ,230 | 5,547 | 1 | ,019 | ,581 | ,370 | ,913 |
| Diagnosis (PMP, no neoplastic cells/colorectal, mesothelioma, others) | 2,090 | ,234 | 79,463 | 1 | ,000 | 8,085 | 5,106 | 12,801 |
| Gender (male/female) | -,471 | ,172 | 7,455 | 1 | ,006 | ,625 | ,445 | ,876 |
| Age | ,000 | ,000 | ,027 | 1 | ,870 | 1,000 | 1,000 | 1,000 |
| ASA score | ,180 | ,128 | 1,974 | 1 | ,160 | 1,198 | ,931 | 1,540 |
| PCI | ,035 | ,008 | 16,708 | 1 | ,000 | 1,035 | 1,018 | 1,053 |

**Goodness-of-fit:**

For each variable, the generalized r-squared value below is shown as a goodness-of-fit value in estimating to what extent the variance in the dependent variable can be explained by the variance of the independent variable.

**Gender**

R2 = 0.024


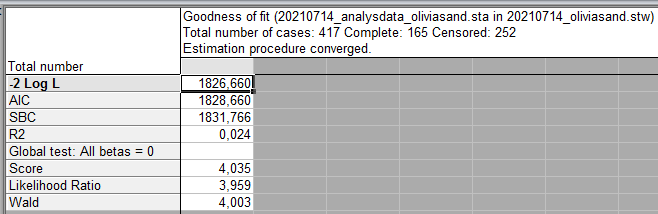


**Age**

R2 = 0.002


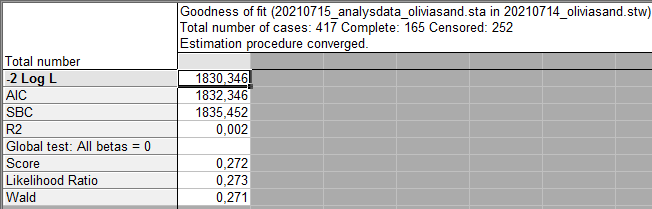


**BMI**

R2 = 0.031


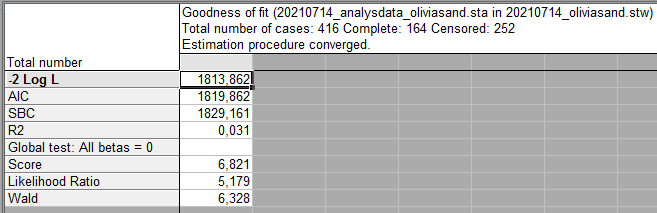


**Comorbidity**

R2 = 0.002


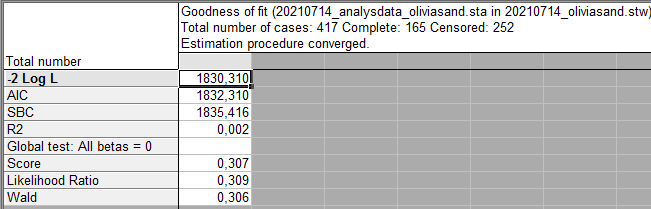


**ASA score**

R2 = 0.023


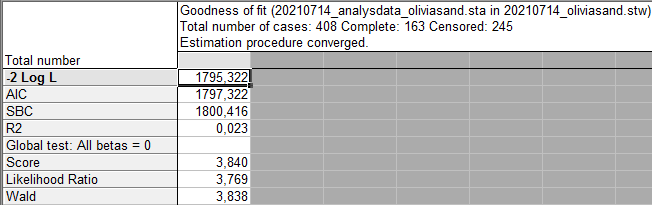


**Diagnosis**

R2 = 0.531


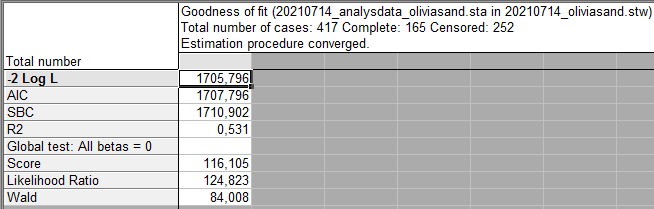


**CC score**

R2 = 0.011


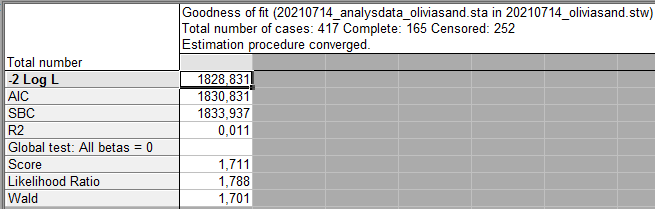


**PCI**

R2 = 0.05


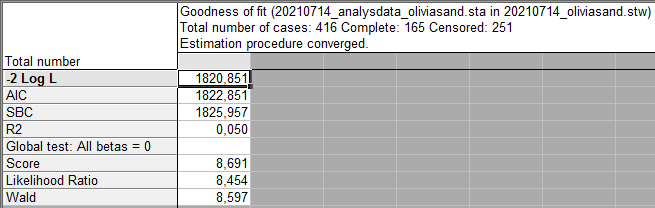


**Liver resection**

R2 = 0.001


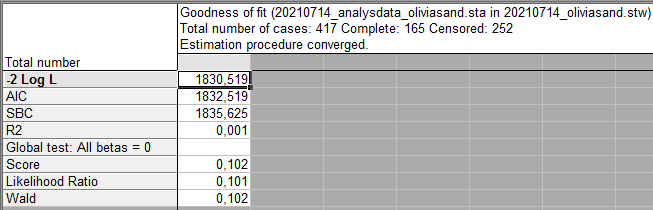


**Splenectomy**

R2 = 0.003


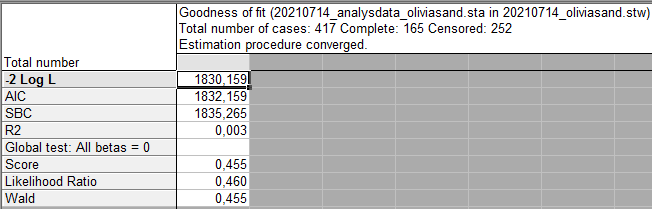


**Complications**

R2 = 0.072


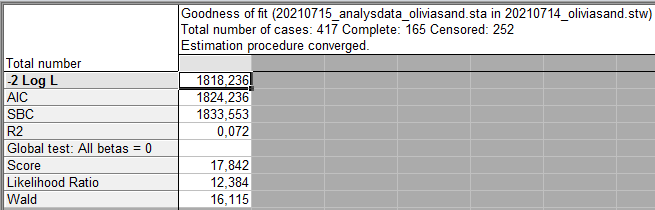


**Overall model goodness-of fit:**

R2 = 0.638


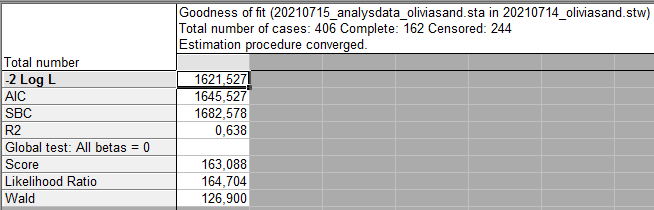


**Assumption of proportional hazards:**

We performed both a visual test as well as a statistical test on the proportional hazards assumption for the Cox regression which is presented below. The deviation from the proportional assumption was interpreted as minor for the complication variable and in view of the large sample size and after consultation with our statistical advisor we believe that use of a proportional hazards method was justified in this study.


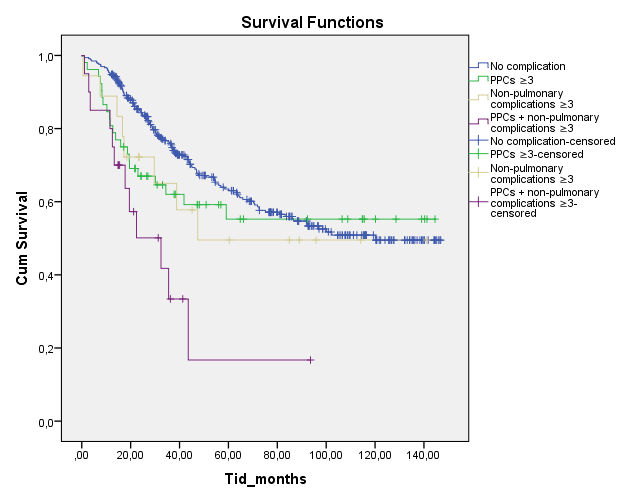


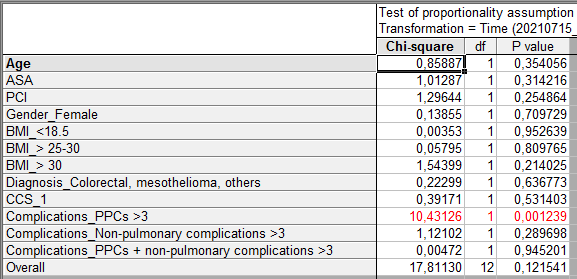

Supplement: S1 Appendix — (DOCX) [file pone.0261852.s001.docx]
