# Supplementary material for: Severe pulmonary complications after cytoreductive surgery and hyperthermic intraperitoneal chemotherapy are common and contribute to decreased overall survival
Source: PLoS One. 2021 Dec 28;16(12):e0261852. doi: 10.1371/journal.pone.0261852 (PMC8714091; doi:10.1371/journal.pone.0261852)
Supplement: Attachment — Submitted filename: Response to Reviewers.docx [file pone.0261852.s002.docx]

Dear Dr. Chen,

Thank you for giving us the opportunity to submit a revised manuscript entitled “Severe pulmonary complications after cytoreductive surgery and hyperthermic intraperitoneal chemotherapy are common and contribute to decreased overall survival” to *PLOS ONE.* We appreciate the time and effort that you have spent to provide feedback on our manuscript.

Below you will find our response to each of the points raised in the email.

1. *No uncommon abbreviation or acronyms in the Title, please! What is HIPEC?*

Thank you for pointing this out. We have now made revisions according to the guidelines of PLOS ONE and spelled out the acronym hyperthermic intraperitoneal chemotherapy. We appreciate that you made us aware of this error.

2. *Please also do English editing in the main text.*

Thank you for your comment. With the help of a native English reviewer, we have edited the main text to improve the text and we believe that this version of the manuscript is of higher quality. Please find the changes made to the manuscript in “Revised manuscript with tracked changes”.

3. *Please ensure that your manuscript meets PLOS ONE's style requirements, including those for file naming.*

We appreciate your comment. In the revised edition of the manuscript please find that we have changed all level 1 headings to bold type with an 18 pt font, all level 2 headings to bold type with a 16 pt font, changed to bold type for all table and figure titles and revised the names of the two figure files and the manuscript file. We have also made sure that the text is double-spaced.

4. *We note that the grant information you provided in the ‘Funding Information’ and ‘Financial Disclosure’ sections do not match.*

*When you resubmit, please ensure that you provide the correct grant numbers for the awards you received for your study in the ‘Funding Information’ section.*

Thank you for making us aware of this error. The information provided in the “Funding information” section now matches the information provided in the “Financial Disclosure”. For clarity, we received funding from the Swedish Cancer Society (Cancerfonden) grant/award number 160411 and Wilhelm Graf was the one who received this research grant.

5. *Please amend either the abstract on the online submission form (via Edit Submission) or the abstract in the manuscript so that they are identical.*

We very much appreciate your thoroughness in noticing this. The abstract on the online submission has now been revised to completely match the abstract in the manuscript.

We look forward to hearing from you in due time regarding our submission and to respond to any further questions and comments you may have.

Sincerely,

Olivia Sand, Aug 9, 2021
