# Supplementary material for: Severe pulmonary complications after cytoreductive surgery and hyperthermic intraperitoneal chemotherapy are common and contribute to decreased overall survival
Source: PLoS One. 2021 Dec 28;16(12):e0261852. doi: 10.1371/journal.pone.0261852 (PMC8714091; doi:10.1371/journal.pone.0261852)
Supplement: Attachment — Submitted filename: Response to Reviewers.docx [file pone.0261852.s003.docx]

Dear Dr. Chen and reviewers,

We thank you for your valuable comments and for the opportunity to submit a revised manuscript entitled “Severe pulmonary complications after cytoreductive surgery and hyperthermic intraperitoneal chemotherapy are common and contribute to decreased overall survival” to *PLOS ONE.* We appreciate the time and effort that you have spent to provide feedback on our manuscript.

Below you will find our response to each of the points raised in the email.

1. *Please review your reference list to ensure that it is complete and correct. If you have cited papers that have been retracted, please include the rationale for doing so in the manuscript text, or remove these references and replace them with relevant current references. Any changes to the reference list should be mentioned in the rebuttal letter that accompanies your revised manuscript. If you need to cite a retracted article, indicate the article’s retracted status in the References list and also include a citation and full reference for the retraction notice.*

The reference list has been reviewed as requested. No article in the reference list has been retracted.

2. *Have the authors made all data underlying the findings in their manuscript fully available?

   The* [*PLOS Data policy*](http://www.plosone.org/static/policies.action#sharing) *requires authors to make all data underlying the findings described in their manuscript fully available without restriction, with rare exception (please refer to the Data Availability Statement in the manuscript PDF file). The data should be provided as part of the manuscript or its supporting information, or deposited to a public repository. For example, in addition to summary statistics, the data points behind means, medians and variance measures should be available. If there are restrictions on publicly sharing data—e.g. participant privacy or use of data from a third party—those must be specified.*

*Reviewer #1: Yes*

*Reviewer #2: No*

We would like to clarify our statement regarding data availability once more. It is due to the General Data Protection Regulation (GDPR) and the potentially sensitive and identifying nature of our data that we cannot share our data publicly. The data are however available upon request from registrator@uu.se (reference UU-DsO 2021/106).

3. *Reviewer #1: In the discussion section, line 216 to line 2018, the authors state that "Unfortunately, we could find no previous studies on PPCs in patients with peritoneal metastases using the Clavien-Dindo classification system, thus making it difficult to compare result". I came across another study, using a rather different, but used Clavien-Dindo classification and included postoperative pulmonary complications among other postoperative complications of CRS and HIPEC.

   Sinukumar S, Mehta S, Damodaran D, Rajan F, Zaveri S, Ray M, Katdare N, Sethna K, Patel MD, Kammer P, Peedicayil A, Bhatt A. Failure-to-Rescue Following Cytoreductive Surgery with or Without HIPEC is Determined by the Type of Complication-a Retrospective Study by INDEPSO. Indian J Surg Oncol. 2019 Feb;10(Suppl 1):71-79. doi: 10.1007/s13193-019-00877-x. Epub 2019 Jan 14. PMID: 30886497; PMCID: PMC6397122.

   I do not think that the findings of that study would add much to the current work in consideration, but I think that, for the sake of perfection, the mentioned sentence should be omitted or the other work get discussed.*

*Reviewer #2: (No Response)*

Thank you for your thorough review and for enlightening us on this study. After reading the article, we have modified the section and the suggested article is mentioned in the revised version. However, in the study by Sinukumar et al., they used the CTCAE classification and not the Clavien-Dindo classification but for the sake of completeness, this article is now discussed and included in the reference list.

4. *(Added Dec 7^th^) Thank you for including your ethics statement on the online submission form: "The study was approved by the Swedish Ethical Review Authority in Uppsala, Sweden (reference no. 2013/203). The data were analyzed anonymously and therefore written/oral consent was not required or obtained.". To help ensure that the wording of your manuscript is suitable for publication, would you please also add this statement at the beginning of the Methods section of your manuscript file.*

Thank you for allowing us to make this revision to our manuscript. We have now added this statement at the beginning of the Methods section both in the main manuscript file and in the manuscript with track changes file.

We look forward to hearing from you in due time regarding our submission and to respond to any further questions and comments you may have.

Sincerely,

Olivia Sand, Dec 7, 2021
